# Implementation of automated alerts for last-resort antibiotics with indication review by infectious diseases specialists: impact on antibiotic prescription quality and costs

**DOI:** 10.1007/s15010-026-02894-9

**Published:** 2026-08-12

**Authors:** Siegbert Rieg, Sarah M. Waibel, Roland Giesen, Leonie Kainz, Maximilian Strub, Daniel Hornuss, Matthias C. Müller, Chloé Kaech, Dirk Wagner, Martin J. Hug, Gesche Först

**Affiliations:** 1 Division of Infectious Diseases, Department of Medicine II, Medical Center, Faculty of Medicine, University of Freiburg, Freiburg, Germany; 2 Medical Center, Hospital Pharmacy, University of Freiburg, Freiburg, Germany; 3 Center for Digitalization and Information Technology, Medical Center, University of Freiburg, Freiburg, Germany; 4 Department of Infection Medicine, Medical Service Centre Clotten, Freiburg, Germany; 5 Clinical Pharmacy, Institute of pharmaceutical Sciences, University of Freiburg, Hermann-Herder-Straße 9, 79102 Freiburg, Germany

**Keywords:** Prescription review, Economic impact, Reserve antibiotics, Infectious diseases consultation, Antimicrobial stewardship

## Abstract

**Purpose:**

Infections caused by resistant pathogens represent a global public health challenge. In the current study we aimed to assess the effects on prescription quality and the economic impact of implementing an automated email alert system for last-resort antibiotics, followed by mandatory indication review by infectious diseases (ID) specialists.

**Methods:**

A prospective single-centre economic and processual evaluation study of orders of last-resort antibiotics at a large tertiary care university hospital (2210 beds) in Germany was performed. Orders of ceftazidime/avibactam, ceftolozane/tazobactam, or cefiderocol triggered an indication review by ID specialists with subsequent recommendations to the primary treating physicians. Expected medication costs of continued therapy were compared to projected costs with adherence to ID recommendations.

**Results:**

The spectrum of infections for which last-resort antibiotics were prescribed was broad, with respiratory, intraabdominal, skin and soft tissue infections representing > 60% of underlying entities. Dominant pathogens were *Pseudomonas aeruginosa*, *Klebsiella* spp. and *Escherichia coli*. ID specialists recommended modifications to the antibiotic regime in almost half of the alerts, including switching (21%), discontinuation (14%) and dosage modifications (7%). Potential cost savings by de-escalation to non-last-resort antibiotics or treatment discontinuation were 65,092€ per year (1,549 € per initial last-resort antibiotic prescription). Adherence analysis revealed that further improvement appears feasible, potentially through structured follow-up ID consultations.

**Conclusion:**

Implementation of automated last-resort antibiotic alerts, followed by an indication review by ID specialists, proved to be a valuable tool with respect to prescription quality and economic benefits. Generalisability of this approach should be investigated in non-tertiary care settings. Moreover, future studies should address potential effects on clinical outcomes and antimicrobial resistance patterns.

**Supplementary Information:**

The online version contains supplementary material available at https://doi.org/10.1007/s15010-026-02894-9.

## Introduction

The management of infectious diseases (ID) is becoming increasingly complex. The growing complexity is driven by an aging population with multiple comorbidities, advances in medical care such as the broader use of implantable devices or potent immunosuppressive therapies, and infections caused by antimicrobial resistant (AMR) pathogens.

Infections with resistant or extensively resistant pathogens represent a global public health challenge associated with substantial morbidity and mortality, and increased healthcare costs, highlighting the importance of rational antimicrobial (AM) use to maintain treatment efficacy (1–5). In response to the challenges posed by AMR, Germany has placed particular emphasis on regulatory aspects concerning the introduction of new antibiotics targeting multidrug-resistant organisms (MDRO). Since 2020, an amendment to § 35a of the German Social Code Book V (SGB V) (6) empowers the Federal Joint Committee (G-BA) to designate new antibiotics as “reserve antibiotics”. For these last-resort antibiotics, additional benefit is considered established without the need for supporting studies from the manufacturer. This regulation aims to provide economic incentives for research, development, and market introduction of AM to combat MDROs.

This regulatory relief is accompanied by several obligations for the prescribing institution. Last-resort antibiotics are only to be used for targeted therapy of infections with MDROs for which limited treatment options exist. Pathogen identification with susceptibility testing must be carried out before use. Empirical therapy with last-resort antibiotics is allowed only in exceptional cases. Consultation with an ID specialist (or a comparably qualified physician) is required prior to therapy initiation. Local treatment guidelines must reference the quality assurance requirements for the use of these agents, and implementation should be integrated into antimicrobial stewardship (AMS) measures. Moreover, hospitals are required to establish a local authorisation policy for last-resort antibiotics. Usage and resistance data must be reported to the national surveillance systems AVS (Antibiotic Consumption Surveillance) and ARS (Antibiotic Resistance Surveillance) at the Robert Koch-Institute (RKI). These additional efforts, as well as the high acquisition costs of still patent-protected last-resort antibiotics, are currently not adequately reimbursed within the German hospital system.

Previous studies have shown that comprehensive AMS programs can promote rational antibiotic use, reduce hospital length of stay, and lower costs, particularly antibiotic expenditures (7–11). The management of complex infections and high-risk patient populations exceeds the scope of AMS interventions. While AMS provides essential structural guidance for antimicrobial prescribing, individualised ID expertise remains crucial for diagnostic clarification, indication assessment, and therapeutic optimisation in complicated cases (12, 13).

ID consultation – a key intervention in the current investigation – has been shown to improve the diagnostic and therapeutic management and is associated with better outcomes especially in severe entities such as bloodstream infection due to *Staphylococcus aureus*, Candida or Gram-negative pathogens (14–17). In the context of *S. aureus* bloodstream infection (SAB), a decision-analytic model evaluation demonstrated that ID consultation in US hospitals is a cost-effective strategy associated with improved survival and reduced health care costs. For the German hospital sector, economic evaluations of ID services are scarce. Accordingly, evidence concerning potential beneficial effects of ID consultation in other countries remains limited to certain infections or patient populations (15, 18, 19).

In the present study we set out to investigate clinical scenarios in which last-resort antibiotic agents are prescribed at a large tertiary care centre. We aimed to evaluate the impact of an automated email alert system, triggered by last-resort antibiotic prescriptions and followed by mandatory indication review by ID specialists, on antibiotic prescription quality and associated costs.

## Methods

### Study design and setting

We conducted a prospective single-centre economic and processual evaluation study of an automated email alert system for orders of last-resort antibiotics, as defined by the German Federal Joint Committee (G-BA), at a large tertiary care university hospital (2210 beds) in Germany (Table 1). The key information regarding the infrastructure of the hospital, ID services and AMS are provided in Table 1.

At the time of initiation of the study, three G-BA-specified last-resort antibiotics (cefiderocol, ceftazidime/avibactam, ceftolozane/tazobactam) were included in the local hospital formulary.

**Table 1:** Overview of study centre and local AMS structure according to indicators of the German AMS guideline

Basic information on the study centre		
**German university hospital**		
Acute care beds	2210	
Inpatient cases/year	90,000	
Outpatient cases/year	1,060,000	
**ID services provided at the study centre**		
Local ID department	Since 2002	
Board-certified ID specialists (excluding fellows)	6 FTE	
24/7 availability of on-call ID service	Since 2005	
Mandatory ID consultation for *S. aureus* bacteraemia	Since 2007	
ID consultation service	~ 2700 consultations/years	
**Structural quality indicators (for AMS) according to** [20]		
Multidisciplinary AMS team, appointed and mandated by hospital administration	**Yes**	Since 2015, mandated since 2019
Representation of the AMS team in the hospitals drugs and therapeutic committee.	**Yes**	Since 2019
At least 2 annual AMS-team meetings	**Yes**	Since 2021
Strategic report hospital management including quantitative objectives and selected performance indicators	**Yes**	Since 2024
Written in-house preanalytical requirements for microbiological samples	**Yes**	
Antimicrobial consumption data available at least annually for the major antibiotic classes as well as for total antimicrobial use, stratified by clinical department or aggregated departments and ward type	**Yes**	Since 1998
Proportion of oral/parenteral AM prescribing available at least annually for the major antibiotics, stratified by clinical department or aggregated departments and ward type	**Yes**	Since 2018
Reporting of AMR resistance rates, *C. difficile* incidence, nosocomial sepsis/bacteraemia rates for clinical isolates available annually	**Yes**	Since 2011
Local Antimicrobial guidelines, electronically available	**Yes**	Since 2021, regularly updated
Electronically available clinical decision support tools for AM use in accordance with local guidelines.	**No**	
Antimicrobial formulary.	**Yes**	Since 2021
Classification of standard and reserve AM within the formulary	**Yes**	Since 2021
Surgical antimicrobial policy	**Yes**	Since 2006
Local recommendations on iv to oral switch of AM therapies	**Yes**	Since 2021
AMS prescriber education provided	**Yes**	Since 2021
Regular AMS ward rounds	**Yes**	Since 2021 on selected wards
Selective reporting of antimicrobial susceptibility testing results	**Yes**	Since 2021

*AM* antimicrobial, *AMR* antimicrobial resistance, *AMS* antimicrobial stewardship, *FTE* full time equivalent, *ID* infectious disease

The study is structured into three time periods: the pre-intervention period, the wash-in period, and the intervention period

### Pre-intervention period

This period was intended to determine the average duration of therapy with last-resort antibiotics in the absence of an alert system. For this purpose, we retrospectively analysed all prescriptions of the three last-resort antibiotics specified by the Federal Joint Committee (G-BA) from the date of market approval of each respective agent (ceftolozane/tazobactam: September 18th 2015; ceftazidime/avibactam: June 24th 2016; cefiderocol: April 24th 2020) until May 31st 2023. Only treatment courses without ID consultation during this pre-intervention period were included in the analysis. Treatment duration was extracted from the medical records and analysed descriptively (mean, median, interquartile range [IQR], minimum, maximum). The results of this analysis were used as baseline to enable the calculation of the mean costs of a course of a last-resort antibiotic therapy.

### Wash-in period

The wash-in period comprised the time during which the initial alert systems directed to the ID service was developed and piloted. On June 7th 2023, first automated email alerts were introduced and subsequently optimised. Since May 7th, 2024, the alert is fully implemented. No data from this period were included in the final analysis.

### Intervention period

**Fig. 1:**
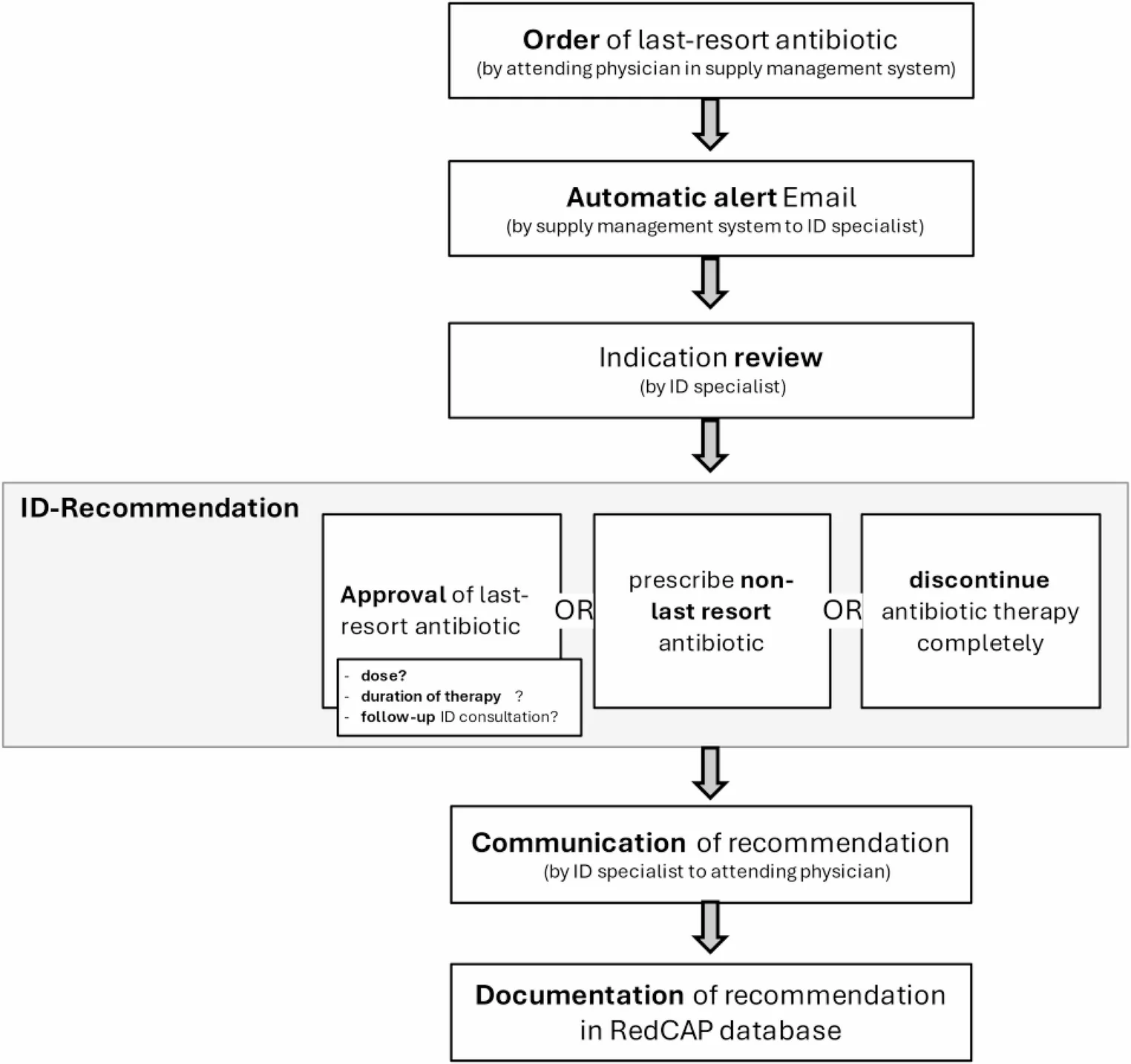
Schematic overview of the intervention

During the intervention period (beginning in June 1st 2024), the alert system for last-resort antibiotics was fully implemented (Fig. 1).

### Alert system

Orders for the three aforementioned last-resort antibiotics placed through the hospital pharmacy inventory and supply management system (SAP SE, Walldorf, Germany) required entry of the patient identification number, the name of the prescribing physician, and the respective hospital ward. Immediately upon order entry, the inventory management system automatically generated an email notification to the ID consultation team.

### Indication review process

Upon receipt of the email notification, a senior ID specialist was required to assess the indication for the prescribed last-resort antibiotic. Based on this assessment, a recommendation was issued to the primary treating team and communicated without delay. Recommendations included continuation of therapy (with or without dose adjustment and with a defined duration or scheduled reassessment), de-escalation to an alternative non-last-resort antibiotic regimen, or discontinuation of therapy. All reviews and corresponding recommendations were prospectively documented in a REDCap database (21) for subsequent analysis.

To ensure a prompt initiation of therapy in critical cases, the pharmacy dispensed the last-resort antibiotic without delay. This process ensures that the therapy with a last-resort antibiotic, once prescribed by the treating physician, can be initiated immediately, even if the formal indication review by the ID specialist requires several hours. Furthermore, the process guarantees that every course of therapy involving a last-resort antibiotic is reviewed and documented by an ID specialist at the earliest possible time point.

Since a single package of the last-resort antibiotics typically covers three days of antibiotic treatment at standard dosing, a subsequent order by the treating physician is required if the continuation of the therapy is planned. This repeat order triggers a new alert to the ID specialist, thereby guaranteeing that every course of therapy is re-reviewed and documented by the ID service within a maximum of three days.

For the purpose of the present study, only the initial prescriptions and the subsequent implementation and outcomes of the corresponding ID recommendations were analysed; follow-up prescriptions were not included in the evaluation.

ID recommendations were communicated to the treating physicians using a multimodal approach tailored to the clinical complexity and urgency of the case. This included brief verbal consultations, digital documentation in the patient chart and detailed written reports in the medical record of the patient. Follow-up was ensured through the aforementioned repeat-order alert system.

### Inclusion and exclusion criteria

The present analysis includes prescriptions for adult inpatients. Only indication reviews associated with initial prescriptions (“first-time prescription”) of one of the three by the G-BA specified last-resort antibiotics (cefiderocol, ceftazidime/avibactam, ceftolozane/tazobactam) were included; reviews of follow-up prescriptions were excluded from analysis.

### Intervention analysis

To estimate potential economic benefits, we evaluated all indication reviews and subsequent recommendations of ID specialists carried out as a result of automated email alerts. For each case, expected medication costs under continued therapy (using the pre-intervention average duration of therapy) were compared to projected costs if the recommendation of the ID specialist (discontinuation, switch, or dose modification) was immediately implemented.

Cost calculations were performed based on the following parameters:

1 $$\begin{gathered} {\text{Daily treatment costs }}\left[ \mathrm{\euro} \right]: \hfill \\ {\text{recommended daily dose }}\left( {{\mathrm{RDD}}} \right) \times {\text{direct drug acquisition costs }}\left[ \mathrm{\euro} \right] \hfill \\ \end{gathered}$$

(References: validated RDD definitions according to German AM surveillance system (22, 23) and ABDA hospital acquisition prices as of May 1, 2025 (24) (see Supplement))

2 $$ \begin{gathered} {\text{Costs per course of therapy }}\left[ \mathrm{\euro} \right]: \hfill \\ {\text{daily treatment cost }}\left[ \mathrm{\euro} \right] \times {\text{treatment duration }}\left[ {\mathrm{d}} \right]\left( {{\mathrm{median}}} \right) \hfill \\ \end{gathered} $$

For therapies that were continued with different agents or at an adjusted dose, the corresponding costs were calculated using the same methodology and the pre-intervention median treatment duration was assumed accordingly. The difference between expected costs (continued last-resort antibiotic treatment for full course of treatment) and projected costs (post-recommendation) represented the potential cost savings.

3 $$ \begin{gathered} {\text{Potential cost savings }}\left[ \mathrm{\euro} \right] \hfill \\ {\text{projected costs}}({\mathrm{post}} - {\mathrm{recommendation}})\left[ \mathrm{\euro} \right] \hfill \\ - \,{\text{costs per course of therapy}}({\text{with last}} - {\text{resort antibiotic}})\left[ \mathrm{\euro} \right] \hfill \\ \end{gathered} $$

Implementation adherence was retrospectively assessed by comparing the documented recommendation of the ID specialist to subsequent prescriptions by the primary treating team. A recommendation was classified as implemented if action was taken within 48 h after the email alert (or, for alerts received Friday/Saturday, by the following Monday).

The actual medication costs incurred were calculated using the same method as for potential savings. The actual cost savings were defined as the difference between the expected costs (continued last-resort antibiotic treatment for full course of treatment) and the actual costs after implementation of ID recommendations.

4 $$ \begin{gathered} {\text{Actual cost savings }}\left[ \mathrm{\euro} \right] \hfill \\ {\text{actual costs}}({\text{after implementation of ID recommendations}})\left[ \mathrm{\euro} \right] \hfill \\ - \,{\text{costs per course of therapy}}({\text{with last}} - {\text{resort antibiotic}})\left[ \mathrm{\euro} \right] \hfill \\ \end{gathered} $$

## Results

### Pre-intervention period

During the pre-intervention period a total of 66 initial prescriptions of last-resort antibiotics (ceftazidime/avibactam, ceftolozane/tazobactam, cefiderocol) in 49 patients (median age 62 years (IQR 48–69 years)) were retrospectively analysed, all of which were initiated without prescription review (Table 2). 17 patients had multiple hospitalisations or more than one course of therapy during the pre-intervention period. Ceftazidime/avibactam and ceftolozane/tazobactam were predominantly prescribed. Only three patients received cefiderocol. The detailed distribution of pre-intervention antibiotics is presented in Table 2. The overall median duration of therapy was 10.5 days. Among the three agents, ceftazidime/avibactam had the longest median duration of therapy (12 days).

**Table 2:** Prescriptions of last-resort antibiotics in the pre-intervention period

Initially prescribed last-resort antibiotics	Number of prescriptions (%)	Duration of therapy [d] median (IQR)
Total	66	10.5 (5.0–15.0)
Cefiderocol	3 (5)	3.0 (2.0-6.5)
Ceftazidime/Avibactam	32 (49)	12.0 (4.5–15.0)
Ceftolozane/Tazobactam	31 (47)	10.0 (7.0–14.0)

### Intervention period

In the intervention period (June 1st 2024–May 31st 2025), 42 initial prescriptions (in 32 patients) triggered an automated email alert and subsequent indication review by ID specialists (Table 3). Seven patients had multiple hospitalisations and received more than one course of therapy during the study period. Median age of the 32 patients was 59 years (IQR 50–68 years).

**Table 3:** Characteristics of infections in the intervention period as judged by ID specialists after prescription review

Initially prescribed last-resort antibiotics	number of prescriptions (%)
Total	42
Cefiderocol	1 (2%)
Ceftazidime/Avibactam	18 (43%)
Ceftolozane/Tazobactam	23 (55%)
Type of infection (per course of treatment)	
Respiratory tract infection (RTI)	17 (40%)
Skin and soft tissue infection (SSTI)	5 (12%)
Intraabdominal infection	4 (10%)
Fever of unknown origin (FUO)	2 (5%)
Urinary tract infection (UTI)	2 (5%)
Bacteraemia	2 (5%)
Septic shock	2 (5%)
Other*	5 (12%)
Undefined / no bacterial infection	3 (7%)
ID-recommendation	
Switch to alternative antibiotic(s)	9 (21%)
Discontinuation of therapy	6 (14) %
Continuation of therapy	27 (64%)
Recommendation on dosage: (For LRAB therapies that were recommended to be continued)	
Continue same dose	24 (57%)
Dose adjustment	3 (7%)
Recommendation on duration of therapy: (For LRAB therapies that were recommended to be continued)	
Not yet determinable / re-evaluation after 3 d	14 (52%)
4–7 d	1 (4%)
8–10 d	6 (22%)
> 10 d	6 (22%)

*Other infections include: Catheter-associated infections, postoperative wound infections, endocarditis, infection of hepatic cysts

The most common type of infection according to the ID specialist’s reviews were respiratory tract infections (RTI 40%; 17/42) followed by skin and soft tissue infections (SSTI, 12%; 5/42) and intraabdominal infections (IAI, 10%; 4/42). In five cases (12%) for whom a last-resort antibiotic was prescribed, no bacterial pathogen was identified. Of these, two cases (septic shock and IAI) a last-resort antibiotic was prescribed based on clinical suspicion of infection. (While in three cases (7%) neither a bacterial infection nor a suspicion of infection could be identified (Table 3).

The pathogens detected in patients with last-resort antibiotic prescriptions are listed in Table 4. *Pseudomonas aeruginosa* was the most frequently detected pathogen, identified in 24 treatment courses (57%). Among these, six isolates were classified as extensively drug-resistant (XDR), nine as multidrug-resistant (MDR), two as pandrug-resistant (PDR) and seven were classified as non-MDR (25). *Klebsiella pneumoniae* was detected in four cases (9.5%). Of these, three isolates were MDR. In two additional cases (5%), *K. pneumoniae* was found in combination with *P. aeruginosa* (in one case both pathogens XDR, in one case both pathogens MDR), and in one case together with *Citrobacter freundii* (both pathogens MDR). Other identified pathogens included *Escherichia coli* (1 MDR), *Enterococcus faecium* (2 non-MDR), *Burkholderia cepacia* complex (1 case), and various polymicrobial infections (two cases, in one case at least one pathogen PDR; in one case all detected pathogens MDR). For five courses of treatment (12%) no pathogen could be identified. Notably, neither the detection of non-MDR pathogens, including *E. faecium*, nor a suspected infection without pathogen identification provided a sufficient indication for the use of last-resort antibiotics. This was appropriately addressed in the following ID service recommendations.

**Table 4:** Microbiological findings in the intervention period

Pathogen(s) (per course of treatment)	Number [*n*] (%)	Resistance pattern according to Magiorakos et al. (25)			
		PDR [*n*]	XDR [*n*]	MDR [*n*]	Non–MDR [*n*]
*E. coli*	1 (2%)	–	–	1	–
*K. pneumoniae*	4 (9.5%)	–	–	3	1
*P. aeruginosa*	24 (57%)	2	6	9	7
*K. pneumoniae* + *P. aeruginosa*	2 (5%)	–	1 (both pathogens)	1 (both pathogens)	–
*K. pneumoniae* + *C. freundii*	1 (2%)	–	–	1 (both pathogens)	–
≥ 3 pathogens	2 (5%)	(in 1 case at least 1 pathogen)	–	(in 1 case all detected pathogens)	–
*E. faecium*	2 (5%)	–	–	–	2
*B. cepacia* complex	1(2%)		–	–	1
No pathogen detected	5 (12%)		–	–	–
**Overall**	42	3	7	16	11

*PDR* pandrug-resistant, *XDR* extensively drug-resistant (XDR), *MDR* multidrug-resistant

### Indication review and ID recommendation

In the 12 months intervention period 23 prescriptions of ceftolozane/tazobactam (55%), 18 prescriptions of ceftazidime/avibactam (43%) and one prescription of cefiderocol (2%) were subject to ID prescription review (Table 3). In approximately half of the cases, the ID recommendation was to continue therapy with the same agent and the same dosage. Dose adjustment was advised in three cases (7%), a switch to an alternative non-last-resort antibiotic in nine cases (21%), and discontinuation of antibiotic therapy in six cases (14%).

For therapies in which the ID specialist recommended continuation of the last-resort antibiotic, specific guidance regarding the duration of therapy was investigated. In 14 of 27 cases (51%), based on the available data at the time of the consultation, a definitive duration of therapy could not yet be determined.

### Adherence to recommendations

During the studied period, 18 ID recommendations were issued regarding discontinuation of therapy, switching to an alternative antibiotic, or dose adjustment. Adherence to these ID recommendations within 48 h was subsequently assessed (Table 5). Two cases could not be analysed concerning the adherence to the ID recommendations due to death, respectively transfer to another institution.

Overall adherence to ID recommendations was moderate (50%). Adherence was highest for dose adjustments (50%) and switch to alternative antibiotics (63%), while therapy discontinuation was fully adhered to in only 2 of 6 cases (33%).

**Table 5:** Adherence to ID recommendations in the intervention-period (within 48 h)

	Number of prescriptions	Adherence (*n*, %)	Non-adherence (*n*, %)
Initially prescribed last-resort antibiotic (with ID recommendation)	18*	8 (50%)	8 (50%)
Type of recommendation			
Dose adjustment	3**	1 (50%)	1 (50%)
Switch to alternative antibiotic(s)	9**	5 (63%)	3 (37%)
Discontinuation of therapy	6	2 (33%)	4 (67%)
Type of last-resort antibiotic			
Cefiderocol	1	1 (100%)	0
Ceftazidime/Avibactam	10**	4 (44%)	5 (66%)
Ceftolozane/Tazobactam	7**	3 (50%)	3 (50%)

* two drop-outs due to transfer or death (not represented in table)

**One drop-out each due to transfer or death in these categories (not represented in table)

### Potential cost savings

Based on the alert-triggered indication review with subsequent recommendations provided by the ID specialists, the estimated annual savings potential amounted to 65,092 € (IQR 30,996 − 92,989 €) (Table 6). This translates to an estimated saving of 1,549 € per initially prescribed last-resort antibiotic. The largest cost savings were associated with targeted therapy switches, which was also the most frequently recommended intervention aside from therapy continuation. Since cefiderocol is by far the most expensive of the three last-resort antibiotics studied, implementing the recommendation to switch or discontinue cefiderocol therapy resulted in particularly large potential savings (10,705 €; IQR 5,098 − 15,293 €), even though this agent was prescribed only once during the study period.

**Table 6:** Potential cost savings per year [€]

	Number of prescriptions	potential cost saving [€] (median)	potential cost saving [€] (IQR)	Potential cost saving per initial prescription of LRAB [€/prescription] (median)	Potential cost saving per per initial prescription of LRAB [€/prescription] (IQR)
Initially prescribed last-resort antibiotic (with ID recommendation)	42	65,092	(30,996 − 92,989)	1,549	(738-2,214)
Type of recommendation	18				
Dose adjustment	3	1,437	(684-2,053)	34	(16–49)
Switch to alternative antibiotic(s)	9	30,144	(14,354 − 43,062)	717	(342-1,025)
Discontinuation of therapy	6	33,512	(15,958 − 47,874)	798	(380-1,140)
**Type of last-resort antibiotic**				Potential cost saving per initial prescription with specific LRAB [€/prescription] (median)	Potential cost saving per initial prescription with specific LRAB [€/prescription] (median)
Cefiderocol	1	10,705	(5,098 − 15,293)	10,705	(5,098 − 15,293)
Ceftazidime/Avibactam	18	21,755	(10,360 − 31,079)	1,209	(576-1,727)
Ceftolozane/Tazobactam	23	32,632	(15,539 − 46,617)	1,419	(676-2,027)

Total differences between expected medication costs under continued therapy with the last-resort antibiotic and projected costs if the recommendation of the ID specialist was immediately implemented (discontinuation, switch, or dose modification) per year; (assumptions: immediate adherence to ID recommendation and a further duration of therapy of 10.5 (IQR 5–15 days) (see Table 2)). LRAB = last-resort antibiotic.

### Actual cost savings

Of the 18 recommendations with cost saving potential made by ID specialists (Table 5), eight were implemented within 48 h, resulting in a realised cost saving of 29,966 € (IQR 14,270 − 42,809 €), equalling to cost saving of 749 €/initial prescription of a last resort antibiotic (IQR 340–1019 €/initial prescription). Two patients died or were transferred within the 48-hour window and were therefore excluded from the analysis (theoretical cost saving for those two patients were 5,470 € (IQR 2,605-7,815 €)). Half of the recommendations (*n* = 8) that were included in the analysis were not implemented in a timely manner. An additional cost saving of 29,656 € (IQR 14,121 − 42,365 €) would have been possible if these recommendations had also been promptly followed.

## Discussion

This study evaluated the implementation and the economic impact of an automated alert-triggered indication review by ID specialists for three last-resort antibiotics at a large German tertiary care centre. Key findings of the current study were (i) the spectrum of underlying infections and causative pathogens was broad with respiratory infections and *P. aeruginosa*, *Klebsiella* spp. and *E. coli* representing dominant alert triggers, (ii) in almost half of last-resort antibiotic alerts modifications to the antibiotic regime were recommended by ID specialists, (iii) potential cost savings by de-escalation to non-last-resort antibiotics, treatment discontinuation or dose adjustment were ~ 65,000 € per year, v.) adherence analysis revealed that while some recommendations were promptly implemented, further improvement appears feasible.

To ensure the rational and restrictive use of last-resort antibiotics, Germany has introduced, among other measures, a mandatory prescription review with confirmation of indication by ID specialists. Although such an ID review is already mandated since 2022, approaches for the practical implementation have not yet been described in detail. As demonstrated by the pre-intervention period, internal hospital policies alone appear insufficient to ensure consistent compliance with these requirements – even in a hospital with an established ID division including ID consultation services (constantly operating with three full-time equivalents, handling approximately 60 bed-side consultations per week) and AMS activities, that are implemented for more than five years. In this context, the automatically generated alert system proved to be a reliable mechanism to operationalise the mandatory indication review and its structured documentation.

Of note, in almost half of indication reviews ID specialists recommended treatment modifications or even discontinuation of costly last-resort antibiotics. While dose modifications were recommended in a minority of cases, de-escalation to non-last-resort antibiotics constituted a relevant proportion (one out of five alerts). This might be due to incomplete consideration or misinterpretation of complex resistance testing results by the primary physician or due to the conclusion of the ID specialist that a non-resistant pathogen was the factual causative agent of the infection. Additionally, in one out of seven alerts ID specialists did not share the notion of an invasive infection but instead considered the resistant pathogen as a colonising strain or innocent bystander and recommended complete discontinuation of antibiotic therapy.

In addition to demonstrating the potential of automated alerts and indication review, our study adds real-life data on the spectrum of MDR or XDR pathogens that necessitate last-resort antibiotics use in a large German tertiary care centre. Infections with *P. aeruginosa* were the cause of last-resort antibiotic prescriptions in over half of the cases. Notably, in our setting MDR *Acinetobacter* spp., which cause a substantial burden of infections in other countries, was not prevalent (11).

In the context of rising inpatient healthcare costs, the projected saving of roughly 65,000 € per year are of particular relevance. The reimbursement of last-resort antibiotics by statutory health insurance in Germany is reportedly underfunded (4, 26), leaving hospitals to absorb a substantial portion of the expenses. The potential to save a meaningful share of these costs provides a strong rationale for continued investment in automated alert systems and dedicated ID personnel.

A strength of the current study is the comprehensive retrospective analysis of cases in the pre-intervention period, allowing for estimation of the therapy duration in the absence of indication review and ID consultation. The study also distinguishes between potential savings (assuming full adherence to recommendations) and actual savings (based on real-world implementation), offering valuable insights into both the theoretical and practical impact of the intervention. The one-year observation period captures seasonal and operational variability, ensuring representative results for a large university tertiary care hospital.

Yet, inherent to its design, the study also bears limitations: Within this study only initial prescriptions of last-resort antibiotics were included in the analysis. The possible impact on follow-up prescribing patterns remains unexamined. Theoretical cost calculations were based on the median pre-intervention duration, however individual therapy lengths varied considerably. Cost estimates were calculated using hospital pharmacy acquisition prices valid as of 1 May 2025. Actual costs may vary at the individual hospital level, depending on specific supply agreements with manufacturers. The single-centre design and focus on an ID-driven intervention in a university hospital may limit generalisability, as resource constraints (as for example the availability of dedicated ID specialists) and technological readiness vary in other settings.

The current study was performed at a time in which three last-resort antibiotics were subject to indication review at the study centre. In the meantime, additional agents such as imipenem/relebactam, eravacyclin, dalbavancin, cefepime/enmetazobactam, aztreonam/avibactam, and meropenem/vaborbactam were added to the mandatory prescription review list (for respective date of G-BA resolutions see Supplement). Thus, implementation of a thorough and prompt indication review – in our centre realised by an automated alert – is becoming ever more important. Moreover, increasing numbers of last-resort antibiotic prescriptions are highly likely, as there is a growing patient group with infections caused by resistant and difficult-to-treat pathogens (27, 28) which will likely increase the economic benefit of alert-triggered ID indication reviews.

Future studies should aim to elucidate factors that are associated with non-adherence to ID specialist recommendations and to develop and evaluate targeted strategies such as optimised communication strategies or potentially more restrictive prescription policies to improve adherence and thereby reduce last-resort antibiotic use.

## Conclusion

The continuous emergence of resistant pathogens and increasing financial constraints in healthcare systems highlight the ongoing need for rapid, expert-guided ID interventions to optimise both clinical and economic outcomes. In our setting implementation of automated last-resort antibiotic alerts with indication review by ID specialists proved to be a valuable tool to enhance prescription quality and to secure economic benefits. Further research should investigate the effects on clinical outcomes and antimicrobial resistance patterns, as well as strategies to maximise adherence to recommendations.

## Supplementary Information

Below is the link to the electronic supplementary material.

Supplementary Material 1

## Data Availability

All data supporting the findings of this study are available within the paper and its Supplementary Information.
