# Supplementary material for: Implementation of automated alerts for last-resort antibiotics with indication review by infectious diseases specialists: impact on antibiotic prescription quality and costs
Source: Infection. 2026 Aug 12;54(5):2643–52. doi: 10.1007/s15010-026-02894-9 (PMC13727928; doi:10.1007/s15010-026-02894-9)
Supplement: Supplementary file 1 [file 15010_2026_2894_MOESM1_ESM.docx]

**Supplement**

**Implementation of automated alerts for last-resort antibiotics with indication review by infectious diseases specialists – impact on antibiotic prescription quality and costs**

**Table S1** Last-reserve antibiotics as classified by the G-BA with respective resolution dates [1]

| **Antibiotic** | **Date of G-BA resolution** |
| --- | --- |
| Aztreonam/Avibactam | 20.02.2025 |
| Cefepime/Enmetazobactam | 05.12.2024 |
| **Cefiderocol** | **05.05.2022** |
| **Ceftazidime/Avibactm** | **03.11.2022** |
| **Ceftolozane/Tazobactam** | **15.08.2022** |
| Dalbavancin | 01.11.2023 |
| Eravacyclin | 19.01.2023 |
| Imipenem/Cilastatin/Relebactam | 03.11.2022 |
| Meropenem/Varborbactam | 17.04.2025 |

**Table S2** Daily cost of therapy of antibiotics included in this study

| **Antibiotic** | **App** | **ABDA hospital acquisition prices as of May 1, 2025 [2]** | **price €/g** | **ADKA-if-DGI RDD [g] [3]** | **price €/RDD** |
| --- | --- | --- | --- | --- | --- |
| Aztreonam | iv | na | 60 | 6.00 | 360.00 |
| Cefiderocol | iv | 1785,00 €/10,0 g | 178.5 | 6.00 | 1071.00 |
| Ceftazidime | iv | 128,14 €/20,0 g | 5.384 | 6.00 | 53.44 |
| Ceftazidime/Avibactam | iv | 1368,50 €/20,0 g | 68.425 | 6.00 | 410.55 |
| Ceftolozane/Tazobactam | iv | 1088.85 €/10,0 g | 108.885 | 6.00 | 653.31 |
| Ciprofloxacin | iv | 155,56 €/4,0 g | 32.68 | 0.80 | 31.11 |
| Daptomycin | iv | 102,58 €/0,5 g | 217.6 | 0.50 | 102.58 |
| Doxycyclin | iv | 14,61 €/0,5 g | 24.56 | 0.20 | 5.84 |
| Gentamicin | iv | 20,17 €/0,8 g | 21.1875 | 0.24 | 6.05 |
| Meropenem | iv | 429,26 €/12,0 g | 30.06 | 3.00 | 107.32 |
| Piperacillin/Tazobactam | iv | 171,54 €/40,0 g | 3.60375 | 12.00 | 51.46 |
| Vancomycin | iv | 266,25 €/10,0 g | 23.374 | 2.00 | 53.25 |

App = way of application, DDD = defined daily dose, iv = intravenous; na = no official hospital acquisition price available; therefore, the in-house price was used.

**References**

[1] https://amr.rki.de/Docs/Reserveantibiotika_SGB.pdf (assessed on 15.12.2025)

[2] ABDATA Pharma-Daten-Service / IFA GmbH

[3] ADKA-if-DGI. Antiinfektiva-Surveillance Projekt [Internet]. 2025. Wirkstoffliste RDD 2025. Verfügbar unter: https://antiinfektiva-surveillance.de/projekt/
